# Limitations of Water Resources Infrastructure for Reducing Community Vulnerabilities to Extremes and Uncertainty of Flood and Drought

**DOI:** 10.1007/s00267-018-1104-8

**Published:** 2018-09-20

**Authors:** Dena W. McMartin, Bruno H. Hernani Merino, Barrie Bonsal, Margot Hurlbert, Ricardo Villalba, Olga L. Ocampo, Jorge Julián Vélez Upegui, Germán Poveda, David J. Sauchyn

**Affiliations:** 1 Environmental Systems Engineering, University of Regina, Regina, SK Canada; 2 Department of Sociology, University of Regina, Regina, SK Canada; 3 Watershed Hydrology and Ecology Research Division, Environment and Climate Change Canada, Saskatoon, SK Canada; 4 Johnson-Shoyama Graduate School of Public Policy, University of Regina, Regina, SK Canada; 5 Instituto Argentino de Nivología, Glaciología y Ciencias Ambientales (IANIGLA), CCT-CONICET-Mendoza, Mendoza, Argentina; 6 Universidad Autónoma de Manizales, Manizales, Colombia; 7 Department of Civil Engineering, Universidad Nacional de Colombia sede Manizales, Manizales, Colombia; 8 Department of Geosciences and Environment, Universidad Nacional de Colombia, Sede Medellín, Medellín, Colombia; 9 Prairie Adaptation Research Collaborative, University of Regina, Regina, SK Canada; 10 Department of Geography, University of Regina, Regina, SK Canada

**Keywords:** Adaptation, Agriculture, Climate extremes, Climate uncertainty, Vulnerability, Water resources

## Abstract

Debate and deliberation surrounding climate change has shifted from mitigation toward adaptation, with much of the adaptation focus centered on adaptive practices, and infrastructure development. However, there is little research assessing expected impacts, potential benefits, and design challenges that exist for reducing vulnerability to expected climate impacts. The uncertainty of design requirements and associated government policies, and social structures that reflect observed and projected changes in the intensity, duration, and frequency of water-related climate events leaves communities vulnerable to the negative impacts of potential flood and drought. The results of international research into how agricultural infrastructure features in current and planned adaptive capacity of rural communities in Argentina, Canada, and Colombia indicate that extreme hydroclimatic events, as well as climate variability and unpredictability are important for understanding and responding to community vulnerability. The research outcomes clearly identify the need to deliberately plan, coordinate, and implement infrastructures that support community resiliency.

## Introduction

Water-related infrastructure employed and constructed in rural and agricultural communities often emerge from the necessity to improve availability, predictability, and timeliness of water access for producing high yield crops and livestock (Loucks and van Beek 2017). Such systems reduce vulnerability to water-related climate extremes in that they serve as both storage of, and access to, water in times of droughts and as protection in times of floods. Rural and agricultural communities tend to collectively develop and implement water infrastructure as adaptations for the support and pursuit of economic and subsidence activities (Peñalba et al. 2012; Smit and Wandel 2006).

Within the context of a changing climate and the focus on adaptive approaches for increasing community and infrastructure resiliency (the ability of social and physical structures to withstand change and stressors, like flooding, while maintaining functionality), the capacity of current and emerging water resources infrastructure designs to adequately achieve the goals of increased resiliency is unknown. Of particular concern are incidents of climate extremes, herein referring to floods and prolonged drought events. In hydrological engineering, precipitation events are formally defined and quantified in terms of intensity, duration, and frequency (IDF). In addition to changes and trends in IDF for a given geographical space, the extent of precipitation events, that is how large and wide spread a rain or snow storm may be, also affects the scale and potential impacts that water may have.

In Canada and South America, the incidence of floods and droughts is changing over time, creating uncertainty in the design and implementation of adaptive water resources infrastructure that have traditionally relied upon historically derived IDF and extent data (Chaney et al. 2015; Sunyer et al. 2014; Zhang et al. 2014). Climate projections indicate that over the next 30 years, the Canadian and South American study regions described herein will experience ever decreasing water supply from snowpack (Carmona and Poveda 2014; Boninsegna and Villalba 2007; Schindler and Donahue 2006). These three regions were selected as part of the Vulnerability and Adaptation to Climate Extremes in the Americas (VACEA) research project because all are “*rural and agricultural, and characterized by communities and economic activities that are sensitive to deviations in climate from normal conditions and to extreme events*” (Sauchyn and Santibañez 2010). Climate projections for agriculturally productive regions of the Canadian Prairies, Argentina’s wine region, and Colombia’s coffee region, all of which are landlocked interiors, indicate an increasingly uncertain future in terms of water accessibility, availability, and predictability (IPCC 2014; Magrín et al. 2014; Lapp et al. 2012). Some results of a changing climate are expected to include more frequent spring flooding, shifts in growing season, and changes in the hydrological cycle with snowmelt beginning earlier in the growing season (Araneo and Villalba 2015; McMartin and Hernani Merino 2014). Such shifts in climate conditions affect both physical and social structures such that more proactive water management is required to ensure that the rural communities involved with the current research can access sufficient water to support and sustain agricultural production as well as community needs (McMartin et al. 2017; McMartin and Hernani Merino 2014).

This paper addresses the research question, *what is the contribution of infrastructures in the studied regions for reducing community vulnerability to climate change, their limitations, and the opportunities to overcome these limitations*. By comparing the use and management of water resources in selected agricultural regions in Argentina, Canada, and Colombia, our objective is to evaluate current agricultural infrastructure as adaptations (systems that enhance resiliency of communities and agricultural producers)—or maladaptations (systems that exacerbate problems rather than serving their designed problem solving purpose)—in terms of capacity and flexibility to maintain and protect agricultural economic activities, livelihoods, and communities in a changing climate (Table 1).

**Table 1:** Comparing aspects of technological adaptation among the three selected agricultural regions

	Argentina	Canada	Colombia
Technological adaptation	Infrastructure supporting water accessibility includes reservoirs, animal watering, and domestic storage and irrigation canals and conveyance systems	Irrigation systems designed with water-smart equipment, timing and apportioning controls, and high efficiency, low-pressure nozzles and sprinklers	A few coffee farmers have implemented irrigation systems as a resiliency measure
Addressing uncertainty	Evaluating impacts on economic development practices and outcomes related to highly sensitive cropping regimes affected by changing climate and extreme climate events	Investments in research related to proactive solutions to climate extremes and impacts on infrastructure	Improving decisions and building processes to reduce infrastructure development in areas of growing uncertainty and instability
Engineering design	No information available	Building Code and national standards set by the National Research Council and similar government agencies; provincial jurisdiction over adoption of the Code or preparation of regional-specific amendments and increased stringency	Flood and erosion control infrastructure designs adapted to changing IDF of water-extremes, including construction of landslide structures (erosion control), rural aqueducts, and reforestation of steep hill slopes
Engineering design and agricultural producer response	Technologies are not necessarily changing, but the economic activity is shifting to accommodate climate and adapting to recognize changes in water availability and ideal growing conditions	Practices of ‘minimum tillage’ that does not disturb the soil as much as previous practices, allowing moisture to be retained; where economically viable, irrigation is developed, with higher efficiency systems used and higher value crops grown	Coffee plantations are moving farther up the mountain with land at lower elevations being converted to fruit crops and sugar cane; some producers shifting main economic activity from coffee agriculture to ecotourism farms
Engineering design and maladaptation	Pre-existing infrastructure was not designed for flood control and often fails under high intensity and duration precipitation events and may create more devastating outcomes than if no infrastructure had been in place at all	Pre-existing infrastructure was not designed for flood control and often fails under high intensity and duration precipitation events and may create more devastating outcomes than if no infrastructure had been in place at all	Pre-existing infrastructure was not designed for flood control and often fails under high intensity and duration precipitation events and may create more devastating outcomes than if no infrastructure had been in place at all

## Methods

We employ a vulnerability assessment model, whereby exposure and sensitivity to climate extremes (primarily flood and drought) and adaptive capacity are investigated for selected rural agricultural communities, and then re-evaluated in the context of projected climate changes (Sauchyn et al. 2016). Participatory research provides further insight via interviews and focus group meetings with local stakeholders and government officials for the assessment of community vulnerability and local governance. Included in the analysis are presentations and interpretation of potential impact of current and projected future climate conditions and variability for the three study regions. Within this presentation is the anticipated change in the IDF of precipitation events that must be planned for and managed by existing or adaptive infrastructure.

Significant challenges in performing rigorous and predictive analyses of water-related extreme climate events for many regions include: (1) considering the return period of extreme events as a static concept, which lurks behind the assumption of a stationary climate, and (2) lack of access to data sets containing high-quality, long-term climate, and meteorological data within a time resolution appropriate for producing high-quality predictive information that can be acted upon. We describe the physical and regional environments and water resources in each study area, as well as past climate trends and future projections. The climate trends and projections provide context to the anticipated challenges and opportunities for adaptation and responsiveness to climate extremes in each study area. Our research method combines collection and categorization of primary data collected directly from community and environmental sources, and secondary data derived from published and publicly accessible sources to identify current and planned climate adaptation processes and designs in each study region. Those data are examined to evaluate how adaptive infrastructure for water management can function under changing climate conditions (i.e., their adaptations or maladaptations per projected climate extremes) and climate uncertainties (i.e., their capacity and flexibility sufficient to respond to climate extremes).

The three regions chosen are all representative of rural, agricultural areas characterized by similar communities and economic activities that are considered to be sensitive to deviations from normal climate conditions and to extreme climate events, despite the differences in the primary agricultural commodities produced in each region. The design overall program of research recognizes the similarities and differences among the selected river basins such that “*a multi-national comparative study of the human and environmental dimensions of the impacts and adaptive responses to short-term climate variability and extreme events”* is both possible and meaningful (Sauchyn and Santibañez 2010). The most economically active and productive agricultural regions of Argentina, Canada, and Colombia are expected to be subject to the impacts of changing climate, primarily in terms of accessibility and timeliness of water for cropped systems (Kraemer 2015; Turbay et al. 2014). The agricultural crops most commonly grown in the three study regions include vegetables and grapes, cereals and oilseeds, and coffee beans, respectively (Kraemer 2015; Poveda et al. 2014).

### Site Descriptions and Water Resources

**Fig. 1:**
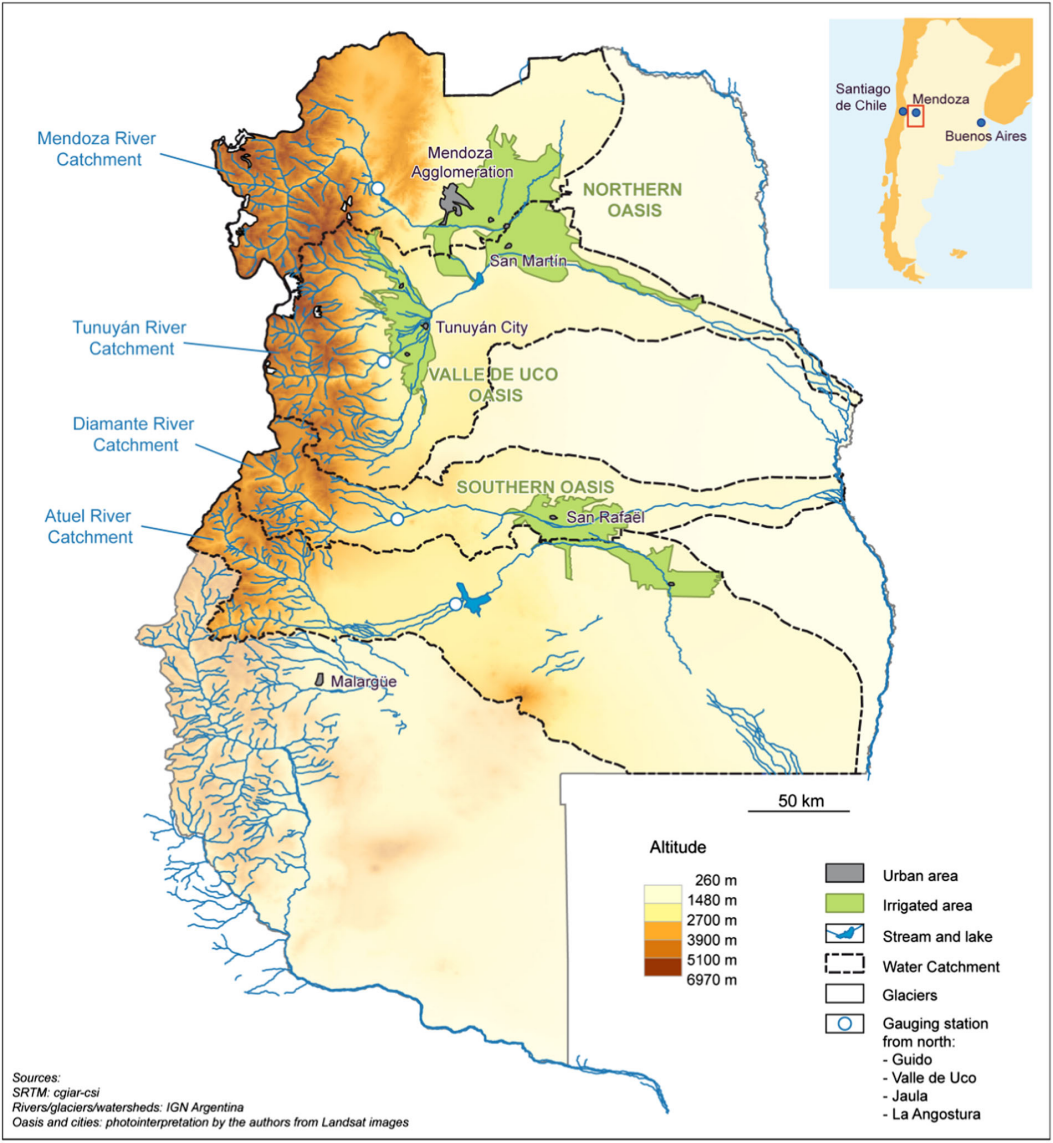
Location of the Mendoza River Basin, Argentina (Delbart et al. 2015)

In Argentina, the Mendoza River basin is the principal irrigated area of the province of Mendoza (Fig. 1). The Mendoza region is characterized by an arid climate situated in the foothills of the Andes mountain range in western Argentina (Bartone 2012). Average annual precipitation in the Mendoza river basin is 224 mm, which has led to high development and dependence on irrigated agriculture (Salas et al. 2012). Irrigated areas are limited to 4–5% of land surface and become oases within an arid landscape dominated by shrubs, grasses, and primary agricultural activities limited to forage for goats and some cows. Across the Mendoza agricultural region, agricultural crops are irrigated by a combination of runoff from the Mendoza and Tunuyan Rivers and, in lower volumes, groundwater (Schlüter and Norrild 2015; Salas et al. 2012). There remain vulnerabilities to water scarcity in terms of timing, imbalance between precipitation and evapotranspiration rates, and anticipated decreases in streamflow in the Mendoza River as snowpack and glaciers in the Andes decline (Villalba et al. 2016; Masiokas et al. 2013; Salas et al. 2012; Montaña 2008).

**Fig. 2:**
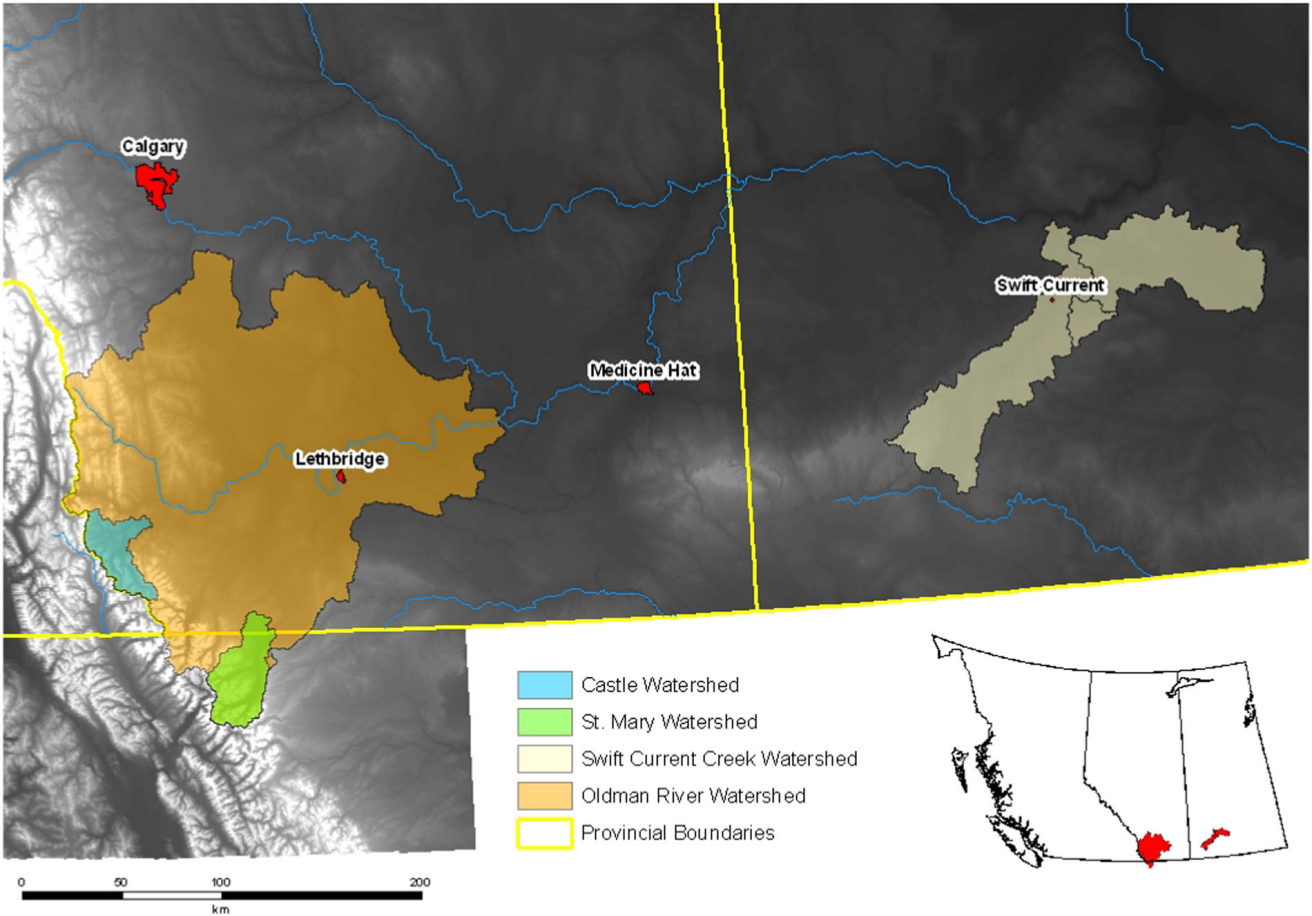
Location the Swift Current Creek and Oldman River Watersheds, Canada (Kienzle, University of Lethbridge, Canada)

The Canadian watersheds included in this analysis are the Swift Current Creek and Oldman River watersheds, respectively (Fig. 2). These agricultural watersheds receive spring melt runoff from the Rocky Mountains (Oldman), as well as snowmelt and limited summer rainfall, in addition to drawing upon reservoir storage to support irrigated cropland to maintain high agricultural productivity focused on cereals, oilseeds, and livestock (primarily cattle) (Thiessen-Martens et al. 2015). With low average annual total precipitation (367 mm; 247 mm as rainfall) in the Swift Current Creek Watershed, the region is particularly vulnerable to drought (Wittrock 2012). The Oldman River watershed in AB is more naturally adapted to high agricultural productivity being both closer in proximity to the Rocky Mountains and more anthropogenically adapted through the extensive use of irrigation (McMartin and Hernani Merino 2014). The Oldman River basin receives total precipitation and rainfall of 398 and 263 mm, respectively (Wittrock 2012). With such low precipitation values, these watersheds are more likely to be affected by drought than flood (Wittrock 2012). Thus, the majority of water management infrastructures are designed to store and convey water in support of community and agricultural activities (Sauchyn et al. 2016).

**Fig. 3:**
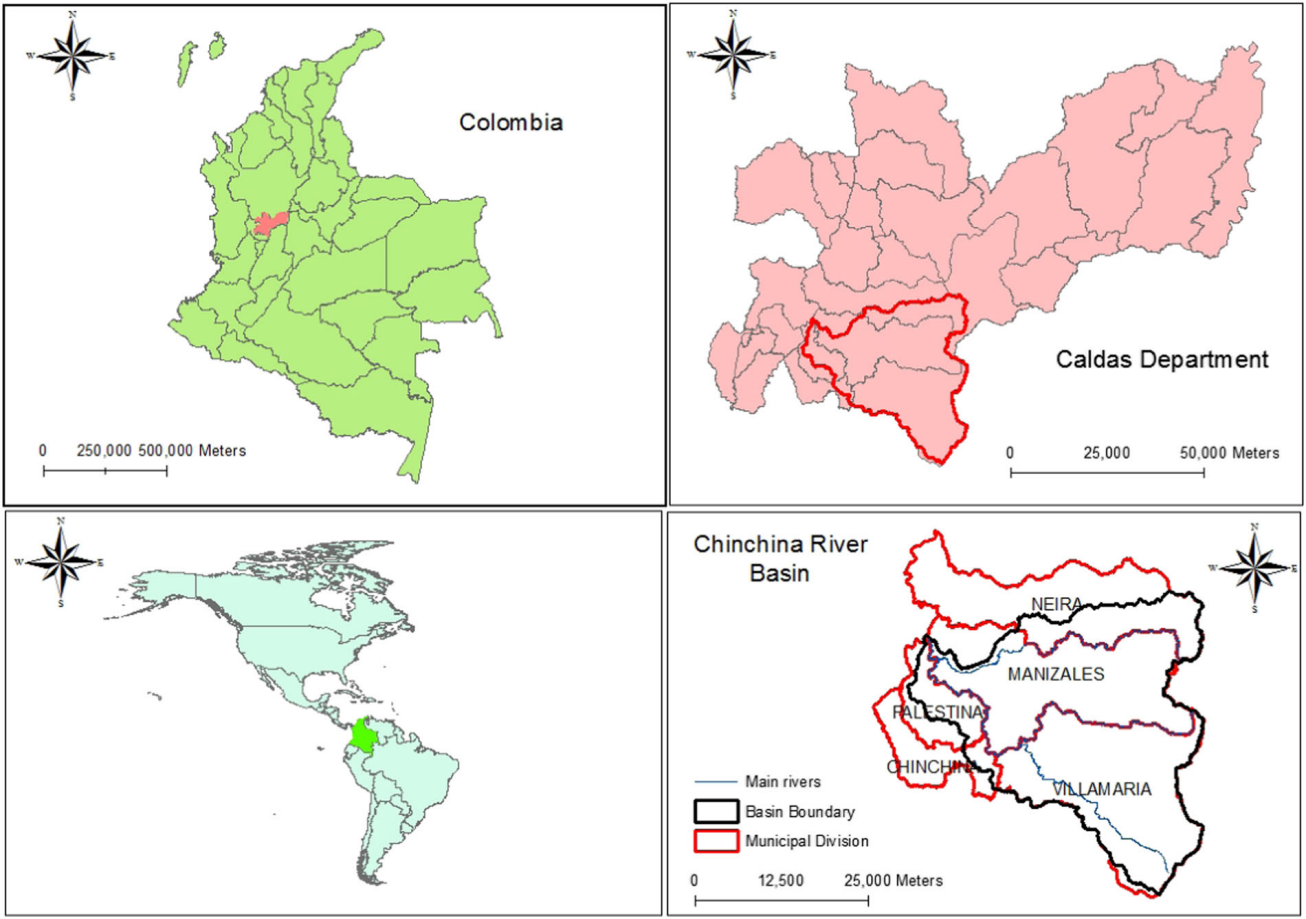
Location of Chinchiná river basin in Columbia (Vélez, National University of Colombia, Manizales)

The Colombian study region is the Chinchiná River Basin (Fig. 3) with an area of 1052 km^2^ and located in the state of Caldas in the Central Andes. The altitudinal gradient extends from 5286 m in the Los Nevados National Natural Park to 860 m at the mouth of the Cauca River. In this region, there are three snowy volcanoes (Ruiz, Santa Isabel, and El Cisne), moorland, mountain forests and a complex of lakes and bogs. The glaciers are retreating and their fate over the 2010–2020 decade was questioned by Poveda and Pineda (2009) in terms of whether or not the glaciers would retreat fully by 2020. The landscape above the 3800 m altitude has a low population density with cattle ranches co-existing with logging, mining, tourism, and forestry alongside conservation enterprises (Nates et al. 2012). The coffee zone is located in the mid to lower altitudes of the Chinchiná River basin between 1000 and 1800 m.

### Climate Projections

Numerous studies outline past trends, climate variability, and predicted climate changes over the three study regions. The incidence of extreme drought and flood events across all three is generally projected to increase. Brief summaries of the projected climate shifts as they pertain to subsequent discussions of adaptive practices, infrastructure development, and community vulnerability and local governance are provided.

An evaluation of the Argentinian study region compared the first decade (1960–1970) with the last (2000–2010) (Deis et al. 2015). The results point to mean temperature increases higher than the global average, increases in the number of days with mean maximum temperature higher than 35 °C and number of consecutive days with minimum temperature higher than 20 °C (indicating heat waves), and decreases in number of frost days. Summer rainfall increases were also noted, despite a decrease in total rainfall events, indicating higher intensity precipitation occurring less frequently. Projected climate change for the region includes an increase of 2–4 °C in mean annual temperature, decrease in winter snowfall in the Andes with the consequent lowering of Mendoza River runoff, and rise in summer rainfall in the plains area (Villalba et al. 2016; Deis et al. 2015).

Annual temperature change for the Canadian Prairies region over the instrumental record of approximately the last 100 years shows a warming of 1.6 °C while water level records, and dendrohydrological reconstructions for the past several centuries, indicate recent declines in streamflow for upstream rivers (Sauchyn and Vanstone 2013; Axelson et al. 2009; Sauchyn and Kulshreshtha 2008). Climate models are consistent in projecting a warmer future for the Canadian Prairies, however, precipitation projections are less conclusive with both wetter and drier futures being predicted (Barrow 2009; Lapp et al. 2009; Sauchyn and Kulshreshtha 2008). The key implications of projected climate change on the Canadian Prairies’ water supply include the timing of spring runoff in the Rocky Mountains and increased water demand due to longer, and warmer, summers with changes in mean annual temperature by the 2080 s ranging from 4 to 6 °C and anticipated precipitation changes range from a decrease of 10% to a maximum of 50% (Sauchyn et al. 2010; Barrow 2009).

The statistical analysis of historical data from 1981 to 2010 in the Chinchiná River basin (Ocampo et al. 2014) supports evidence of climate change reflected in increasing mean temperatures by 0.5 °C. The tendency for solar radiation is decreasing and no trends appear to be present for precipitation and relative humidity. Rainfall projections for 2020–2049 demonstrate minimal trends. However, modeling results point to anticipated precipitation between 1175 and 2880 mm (Ocampo and Vélez 2014).

### Adaptation to Water-Related Climate Extremes

Globally, homogeneity of data is a problem, particularly for identifying and quantifying extreme events (e.g., days exceeding a specific threshold) (St. Jacques et al. 2014). Recently much attention has been paid to changes and impacts on major water sources across the Canadian Prairies (Dumanski et al. 2015; St. Jacques et al. 2014) and Colombia (Carmona and Poveda 2014), and western Argentina (Masiokas et al. 2013), focusing on trends and variability in lake and river water levels. Infrastructures that are commonly employed to support efficient and effective water use in drought and, in some cases, to limit hillslope instability that results from indiscriminate watering, in both the Argentinean and Canadian study regions include irrigation systems designed with water-smart equipment, timing and apportioning controls, and high efficiency, low-pressure nozzles and sprinklers. Infrastructure supporting water accessibility includes reservoirs, animal watering and domestic storage (dugouts), and irrigation canals and conveyance systems. We consider these as adaptations to climate, but each is limited in scope, design or construction within the context of water-related climate extremes, such as drought and flood conditions. In Colombia, there are opportunities to design irrigation systems holistically in consideration of changing climate since, at this time, only a few coffee farmers have implemented irrigation systems as a resiliency measure. It is anticipated that such engineering systems will constitute part of the adaptation strategies for Colombia as agricultural producers adapt to the changing societal, economic, and environmental landscapes in which they operate.

Uncertainty is a constant concern in the development of climate projections and the application of those scenarios to community planning and infrastructure development. The coupling of eco-hydrological and agricultural models is a key approach for understanding agro-ecological and land use changes and reducing uncertainty during modeling processes. Uncertainty can be related to both measurements and model structure and assumptions, and is reduced through improved data management and expansion of networks of stream flow gauges and meteorological stations and appropriate use of new engineered systems. The uncertainty related with environmental parameters can be tackled with methodologies available in the scientific literature, such as the generalized likelihood uncertainty estimation (GLUE) (Beven 1992); while model uncertainties are addressed through use of multi-model techniques that narrow the selection of best-fit climate models by choosing only those capable of capturing the seasonal impacts present in historical data. Some uncertainty is irreducible given the complexity and internal variability of the climate system (Deser et al. 2014).

If climate projections converge through some of the techniques described above, it is essential for the outputs to be incorporated into IDF curves and planning scenarios at the municipal, and even individual producer level. The IDF technique must be reviewed and updated to include the changing patterns detected in temperature and expected for rainfall using the future climate scenarios. Of particular importance is the need to have higher resolution maps of future changes in temperature and rainfall due to climate change and deforestation, either from dynamical or statistical downscaling techniques. Through such updates, improved data quality can inform legal frameworks, construction codes and standards, and design of best practices for managing and mitigating flood and drought conditions.

Knowing the behaviors and tendencies of the people responsible for implementing new and adaptive infrastructure, the science and communication of methods, approaches, and models must be, by definition, transdisciplinary (social and natural sciences) and clear. Agricultural producers tend to be relatively conservative in terms of being change leaders. Thus, uncertainty should be addressed fully in the scientific process and engineering design, as well as in communications strategies and decision-making processes undertaken by municipalities and landowners.

As a result of observed climate changes and anticipated climate projections, engineering designs that rely on historically based statistics to create IDF curves are potentially not reliable for the future. Since most adaptive practices are long-term investments (for producers and communities), infrastructure changes are slow to respond to changing climate and the increase in frequency, extent, and intensity of climate extremes. The development of engineering strategies and national, provincial, or regional protocols for the decisions, designs, and construction of infrastructure for mitigating the negative impacts of flood and drought within a region must acknowledge that not every year or every event will conform to the climate projections. Engineering design can be informed by several mechanisms including building codes and standards that are established by national organizations and governments. In Canada, such codes and standards are set by the National Research Council and similar government agencies, but each province has jurisdiction over whether or not to adopt the code or prepare regional-specific amendments and increased stringency. Regardless, it is a political process of negotiation between industry groups and stakeholders to arrive at these standards. Due to this negotiation process, many considerations (cost, safety, ecological integrity, socioeconomic benefit, etc.) are factored into the development and decision-making processes around which standards are adopted and, because of the political process involved, these standards may or may not account for changing climate and projected climate change. In all cases, local authorities (i.e., municipalities) are responsible for implementing these standards and paying for the associated design that factors in the climate conditions with the infrastructure plan.

The research uncovered some examples where engineering design accurately accounted for climate change. In Colombia, flood and erosion control infrastructure designs are adapted to reflect changing IDF of water-extremes, including the construction of landslide structures (erosion control), rural aqueducts, and reforestation of steep hill slopes and water sources. Another scientific response, the precipitation area duration frequency curves used for predicting extreme events have been revised using different climate change scenarios for designing effective disaster risk management strategies. Poveda et al. (2014) observed changes in agricultural producer communities and behavior in order to adapt to the impacts of recent El Niño events, including manual irrigation during drought periods, incorporation of mixed crops with coffee (plantain and walnuts), renewing coffee plantations with drought and plague resistant varieties, and cooperative strategies among farmers during rainy periods.

There remains one important example from the Colombian study site that illustrates the need for changes in the design and operation of hydropower infrastructure throughout the Chinchiná River basin, since structures were designed according to the environmental or minimum flows based on historical normals. Neither the local authorities nor the energy company have adopted the revised climate-informed curves, resulting in infrastructure and construction standards not keeping pace with climatological science and hydrologic projections (Boodoo et al. 2014).

Agricultural producers in all study regions made changes to the engineered systems and infrastructure associated with their agricultural practices, thereby adapting in response to climate change. In Canada, producers are adopting practices of ‘minimum tillage’ that does not disturb the soil as much as previous practices, allowing moisture to be retained. Where irrigation is affordable and available, higher efficiency systems are in place and higher value crops are grown.

In Argentina, changes are being experienced in relation to agricultural crops and the ideal conditions for those crops. Some agricultural producers are adopting more efficient irrigation equipment. Vineyards are adjusting the grape varietals as well as the locations in which certain varietals are grown in direct response to changing weather patterns and shifts in climate related to precipitation and extreme temperatures. The technologies are not necessarily changing, but the economic activity is shifting to accommodate climate and adapting to recognize changes in water availability and ideal growing conditions.

In Colombia, coffee plantations are moving farther up the mountain with land at lower elevations being converted to fruit crops and sugar cane, with some producers shifting their main economic activity from coffee agriculture to farms for ecotourism. Many Colombian producers adopted traditional coffee growing techniques using banana trees for shade of coffee plants. Again, the agricultural productivity remains but dependence on water availability and adequate temperatures remains a key element for producing ideal growing conditions for coffee, fruit trees, and sugar cane.

Water-related infrastructure requirements and flexibility in design will increase as water resource management becomes more sophisticated and responsive to climate in an effort to maintain food security and agricultural industry stability. In many instances, engineering solutions to improve community and regional resiliency to drought may also be adapted for use in times of flood. Challenges exist in this approach where gravity flow of water is part of the design, for example in irrigation canals. However, even a gravity-flow irrigation canal can be adapted to permit storage of excess water, delay peak-flow and reduce overland flow rates and volumes.

The growth and success of establishing perennial well-rooted crops on hillsides, such as coffee and grapes, aids in slope stability and reduces erosion under flood conditions. The development of dam and reservoir systems provides additional water capacity and can either negate or delay peak flow from impacting downstream infrastructure and lands. The construction and expansion of irrigation canals and rural aqueducts can serve as temporary water storage capacity and reverse-flow management technologies in times of large volume, high intensity precipitation events. In each study region, there is growing capacity for water storage that can serve as both drought and flood infrastructure, despite the primary impetus being focused on drought management. However, pre-existing infrastructure was not designed for flood control and often fails under high intensity and duration precipitation events and may create more devastating outcomes than if no infrastructure had been in place at all. Such “maladaptations” in infrastructure exist, for instance, in terms of maintenance and operation plans (e.g., blocked culverts or overgrown canals) that may not significantly impede performance under drought or scarce water conditions, but can cause increase of vulnerability to land and communities under flood conditions. Thus, a maladaptation can be said to occur when either an action or inaction increases vulnerability.

Through communications with agricultural producers, consideration and projection of historical and anticipated climate regimes, and coordinated design and implementation of infrastructures capable of adequately managing water and protecting against the impacts of extreme water events, communities are engaged, educated, and resilient to negative impacts of changing climate. The limitations of infrastructures lie not only within current design models and understandings of IDF of water events, but also within the intersections of community, livelihoods, and governance of those infrastructures. As engineering systems are revisited and redesigned to reflect climate-adjusted IDF curves and projected extreme water events, so too must the maintenance and operating standards be revised to ensure safe and reliable performance in times of extremes.

In all three study regions, it was noted that physical and engineered adaptation for drought is led not only because of historically dry conditions, but also as response to the intensification of climate variability, being a combination of proactive with reactive actions. In contrast, infrastructural adaptation to address flooding tends to be a response to a great degree to the impacts of flooding on infrastructure, being more reactive than proactive depending on the sector affected (e.g., hydropower, agriculture, fluvial navigation, cattle ranching, or human health).

## Conclusions

Across all three study regions, the data, observations, and agricultural producer experiences all point to changing frequency and intensity of floods and droughts accentuated by climate change. In each, adaptations through infrastructure, as well as behavior and land use management, are taking place despite full understanding or formal incorporation of climate change into legal frameworks, standards, and codes, because municipalities and individual land owners have vested interests in the preservation of the economic drivers in their region.

This research study found that generally, engineering designs of water-related infrastructures (collectively such as reservoirs, irrigation, rural aqueducts, and slope stabilization efforts) are implemented as adaptations to climate, but are not necessarily designed or constructed in the context of climate change and specifically more frequent and more intense climate extremes. Often, these adaptive infrastructures are designed relying on historically based statistics that are potentially not reliable for future design of infrastructure to manage flooding and higher than normal precipitation or extended droughts, nor might they be adequate for addressing adaptation generally due to shifts in IDF of precipitation or flooding events. Because most of these adaptive practices are long-term investments (for agricultural producers, communities, and governments), changes in infrastructure are slow to respond to changing climate and the related increase in IDF of climate extremes.

New engineering designs, codes, and practices will be a fundamental element of adaption to mitigate the impacts of extreme weather in changing climate. Research results reveal, however, that the adoption of new engineered systems and agricultural practices is subject to social and institutional factors that both enhance and constrain the adaptive capacity of rural communities. Planned adaption to climate change requires collaboration among agricultural producer associations, the agricultural industry, non-government organizations, and local and regional government agencies.
